# P-1652. Cognitive performance long after COVID infection: results of a cross-sectional pilot study

**DOI:** 10.1093/ofid/ofaf695.1827

**Published:** 2026-01-11

**Authors:** Kristen Kehl-Floberg, Fauzia Hollnagel, Emily Danzl, Dorothy Farrar-Edwards, Aurora Pop-Vicas

**Affiliations:** University of Wisconsin School of Medicine and Public Health, Madison, Wisconsin; UW Madison, Madison, Wisconsin; University of Wisconsin School of Medicine and Public Health, Madison, Wisconsin; University of Wisconsin School of Medicine and Public Health, Madison, Wisconsin; University of Wisconsin School of Medicine and Public Health, Madison, Wisconsin

## Abstract

**Background:**

Previous studies found neurocognitive deficits in 20-50% of Long COVID patients, although the objectively measured prevalence of these impairments is typically lower than the subjectively reported one. It is not clear whether these deficits are long-lasting. Our main goal was to objectively assess cognitive performance in people with Long COVID years after initial infection versus controls.

**Figure:**
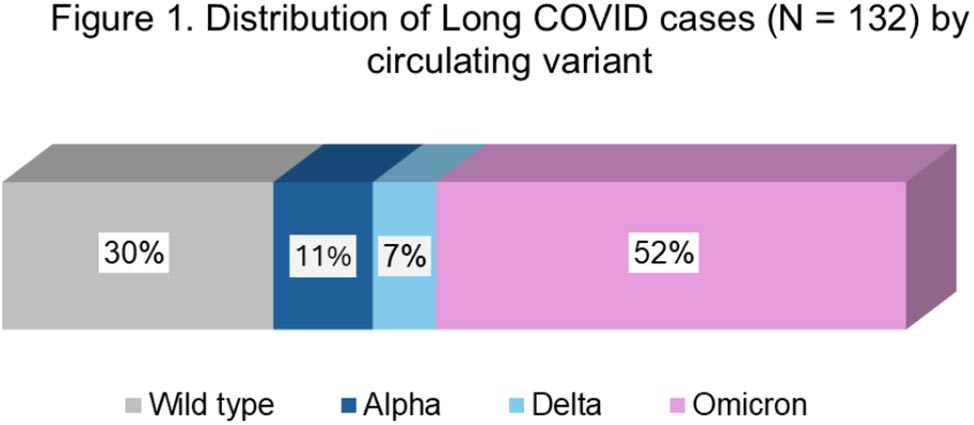

**Figure:**
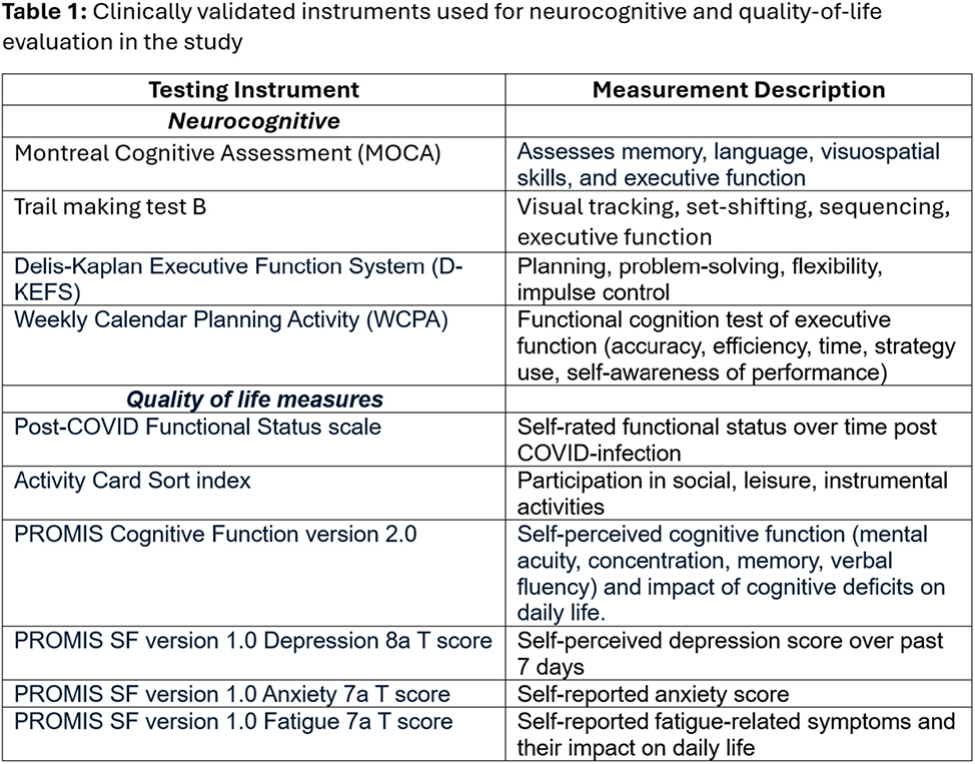

**Methods:**

Study design: cross-sectional pilot enrolling a convenience sample of adults ≥ 18 years old recruited from a large Midwest university and its affiliated state county. Cases: people with at least one persistent post-COVID symptom ≥ 6 months after laboratory-confirmed initial infection. Controls: people without known prior COVID-19 infection. Outcome measures: Cognitive performance, assessed via a complex battery of neuropsychological and functional cognition tests, and quality-of-life variables assessed via patient-report on clinically validated instruments (Table 1). *Statistical analysis:* Univariable and multivariable logistic regression adjusted for the interaction between age and education. A two-sided p-value < 0.05 was considered statistically significant.

**Figure:**
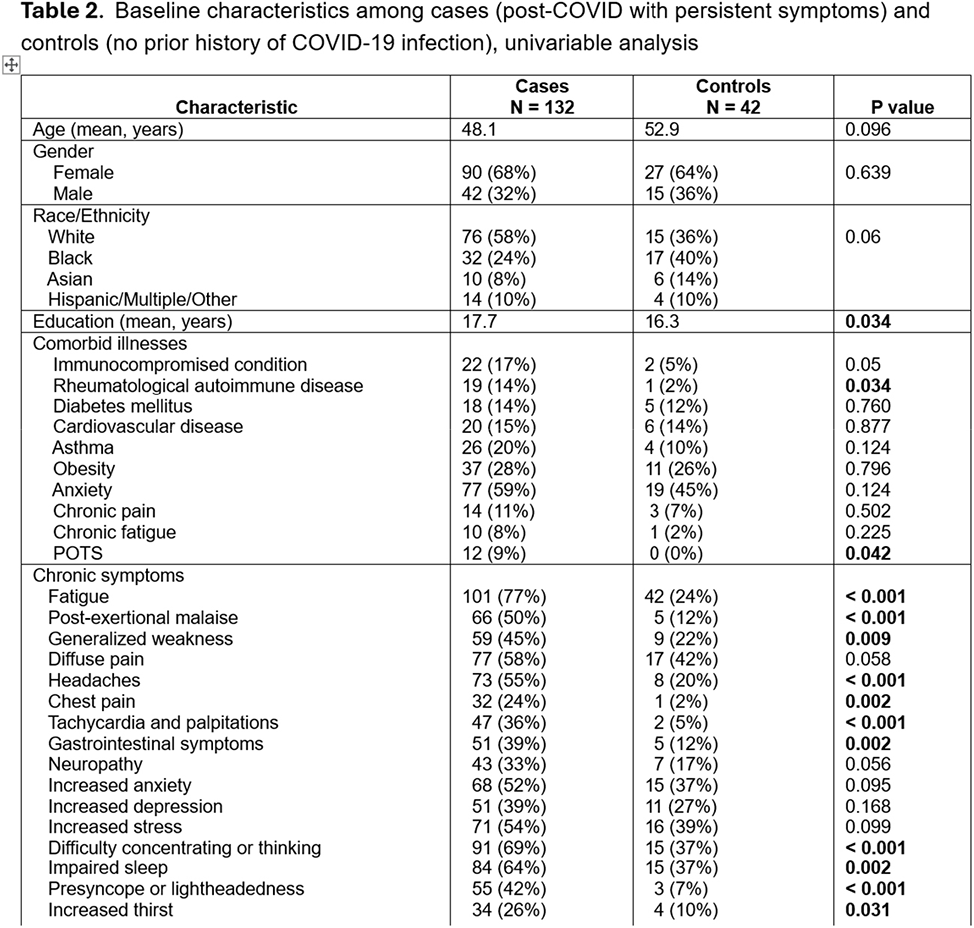

**Figure:**
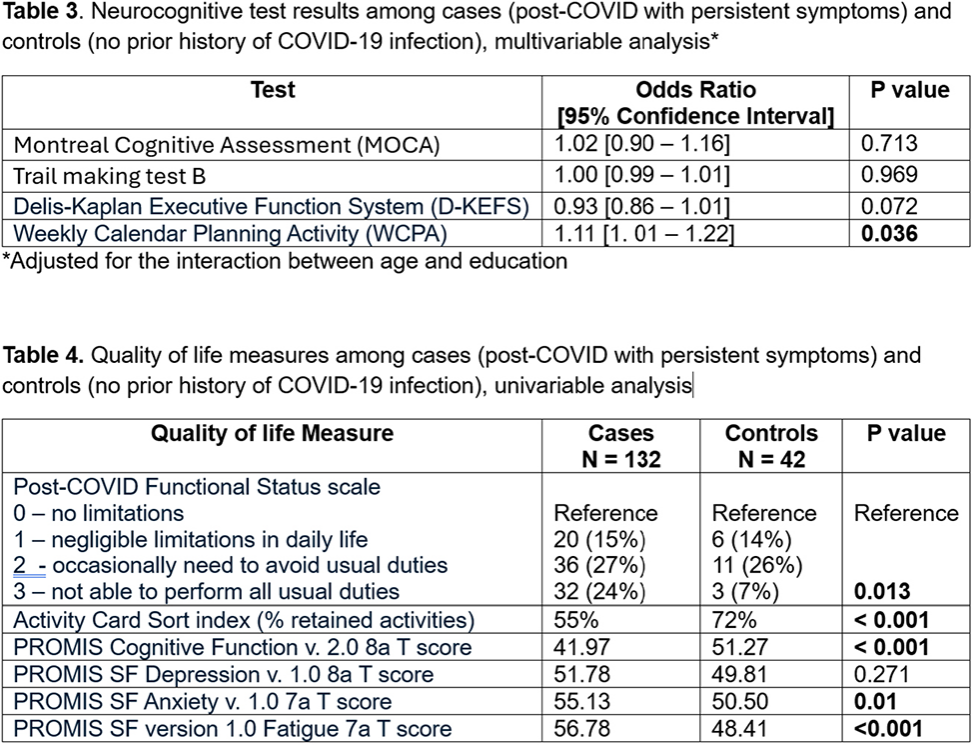

**Results:**

We enrolled 132 post-COVID cases and 42 controls. The average time from initial infection was 2.4 years (range 6 months – 4.8 years). Figure 1 shows the distribution of cases according to the major circulating strain at the time of initial infection. Table 2 shows differences in baseline characteristics among cases and controls. Table 3 shows the neurocognitive testing results for cases and controls. Table 4 shows differences in other quality of life outcomes among cases and controls.

**Conclusion:**

Long COVID participants scored significantly worse on instruments rating self-perceived functional status, percent of retained daily activities, anxiety, fatigue, and cognitive function, suggesting persistently disrupted instrumental activities of daily living and lower quality of life years after initial infection. On most of the objective neurocognitive testing, however, there were no significant differences between Long COVID patients and controls.

**Disclosures:**

All Authors: No reported disclosures

